# Phylogenetic analysis of Chinese sheeppox and goatpox virus isolates

**DOI:** 10.1186/1743-422X-9-25

**Published:** 2012-01-20

**Authors:** Tao Zhou, Huaijie Jia, Guohua Chen, Xiaobing He, Yongxiang Fang, Xiaoxia Wang, Qisai Guan, Shuang Zeng, Qing Cui, Zhizhong Jing

**Affiliations:** 1 State Key Laboratory of Veterinary Etiological Biology, Key Laboratory of Veterinary Public Health of Agricultural Ministry, Lanzhou Veterinary Research Institute, Chinese Academy of Agricultural Sciences, Lanzhou 730046, China

**Keywords:** Sheeppox virus, Goatpox virus, P32 gene, GPCR gene, RPO30 gene, Phylogenetic analysis

## Abstract

**Background:**

Sheeppox virus (SPPV) and goatpox virus (GTPV), members of the *Capripoxvirus *genus of the *Poxviridae *family are causative agents of sheep pox and goat pox respectively, which are important contagious diseases and endemic in central and northern Africa, the Middle and Far East, and the Indian sub-continent. Both sheep pox and goat pox can cause wool and hide damage, and reduce the production of mutton and milk, which may result in significant economic losses and threaten the stockbreeding. In this study, three SPPVs and two GTPVs were collected from China in 2009 and 2011. We described the sequence features and phylogenetic analysis of the P32 gene, GPCR gene and RPO30 gene of the SPPVs and GTPVs to reveal their genetic relatedness.

**Results:**

Sequence and phylogenetic analysis showed that there was a close relationship among SPPV/GanS/2/2011/China, SPPV/GanS/1/2011/China and SPPV/NingX/2009/China. They were clustered on the same SPPV clade. GTPV/HuB/2009/China and GS-V1 belonged to the GTPV lineage. GS-V1 was closely related to other GTPV vaccine strains. GTPV/HuB/2009/China and GS-V1 were clustered with GTPVs from China and some southern Asian countries.

**Conclusion:**

This study may expand the datum for spread trend research of Chinese SPPVs and GTPVs, meanwhile provide theoretical references to improve the preventive and control strategy.

## Background

Sheeppox virus (SPPV), goatpox virus (GTPV) and lumpy skin disease virus (LSDV) comprising Genus *Capripoxvirus *of the Family *Poxviridae *are important aetiological agents to ruminants. They cause sheep pox, goat pox and lumpy skin disease of cattle respectively, which are well-known economically significant animal diseases. Sheep pox and goat pox, classified as notifiable animal diseases by the World Organisation for Animal Health (OIE), are mainly endemic in central and northern Africa, the Middle East, Indian sub-continent, central Asia and parts of P. R. China [1]. In contrast, the endemic geographic range of lumpy skin disease is currently limited to Africa, with sporadic outbreaks occurring in the Middle East [2,3].

Sheep pox and goat pox are characterized by pyrexia, rhinitis, conjunctivitis, excessive salivation and generalized pock lesions in the skin. They may cause the significant damage to wool and hides (can affect as much as 50% of the skin surface) and the production loss of mutton and milk [2]. Furthermore, sheep pox and goat pox may be associated with very high morbidity and mortality rate except indigenous animals. The mortality rate in young animals can exceed 50%. However, in naïve animals, morbidity and mortality can even approach to 100% [4]. As for host preference, it was once a common belief that SPPV only infected sheep while GTPV only infected goats. But now, more researches have supported that the host specificity of SPPV and GTPV strains was not limited to either sheep or goats. Besides, some reports about natural outbreaks may rather reflect that goats may have relatively mild clinical disease when infected with SPPV compared to severe disease in sheep, likewise, sheep may have relatively mild clinical disease when infected with GTPV compared to goats infection [5,6]. Conventional serological test cannot distinguish SPPV, GTPV and LSDV, so identification of these pathogens needs molecular methods such as restriction endonuclease analysis (REA) [7] and polymerase chain reaction-restriction fragment length polymorphism (PCR-RFLP) [8].

P32 gene, GPCR gene and RPO30 gene were used in this work on phylogenetic analysis. P32 corresponds to an envelope protein homologous to P35 protein encoded by Vaccinia virus H3L gene, and locates on the membrane surface of the mature intracellular viral particle [9]. GPCR gene encodes a protein related to the G-protein-coupled chemokine receptor subfamily. The protein has the key structural characteristics of the G-protein-coupled chemokine receptor superfamily, such as seven hydrophobic regions and the cysteine residues in the first and second extracellular loops [10]. RPO30 gene is a homologue of the Vaccinia virus E4L gene, which encodes the 30 kilodalton DNA-dependant RNA polymerase subunit [3]. P32, GPCR and RPO30 genes are highly conserved among capripoxviruses. Sequence information of them can be used in differentiating SPPV, GTPV and LSDV, presenting the genetic relationship among different virus strains [3,8,10]. Here, we cloned and sequenced P32 gene, GPCR gene and RPO30 gene of four SPPV/GTPV isolates mentioned above and another GTPV vaccine strain. Furthermore, the sequencing results were used for phylogenetic analysis by comparing them with various capripoxvirus isolates retrieved from GenBank to elucidate the genetic relatedness of these viruses.

## Results

### Detection of suspicious samples

**Figure 1:**
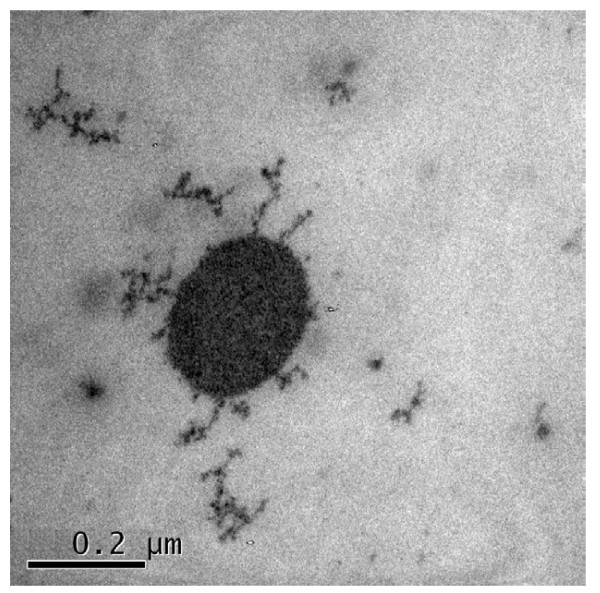
**Electron microscope examination of capripoxvirus (SPPV/GanS/2/2011/China)**.

Suspicious papule/scab samples from each case were used for diagnostic analysis. In brief, expected amplicons of approximate 545 bp were obtained for all samples in PCR test, and the viruses were obtained by cultivated in Vero cells. Then, viruses were passed in Vero cells with 10 ~ 12 times. Concentrated viruses were further observed under transmission electron microscope. The electron microscope examination showed that the viral particles had an appearance of approximate oval, and surrounded by a large number of tubular units (Figure 1).

### Sequence analysis of P32 genes, GPCR genes and RPO30 genes

The complete open reading frames (ORFs) of P32 gene, GPCR gene and RPO30 gene of five capripoxvirus strains were sequenced and subjected to similarity analysis.

The results showed that the five P32 genes of SPPV/GanS/2/2011/China, SPPV/GanS/1/2011/China, SPPV/NingX/2009/China, GTPV/HuB/2009/China and GS-V1 shared very close relationships with other capripoxviruses isolated from different regions (the nucleotide sequence identities were 97.9 ~ 100%, 97.9 ~ 100%, 97.8 ~ 99.9%, 97.3 ~ 99.9%, 97.5 ~ 100%, respectively). SPPV/GanS/2/2011/China and SPPV/GanS/1/2011/China shared the 100% identity with Pune-08 (SPPV, India), TU-V02127 (SPPV, Turkey), Makdhoom (SPPV, India) and Shanxi (SPPV, China). GS-V1 was 99.8% identical to GTPV/HuB/2009/China. GPCR genes of SPPV/GanS/2/2011/China, SPPV/GanS/1/2011/China, SPPV/NingX/2009/China, GTPV/HuB/2009/China and GS-V1 shared 95.2 ~ 99.7%, 95.0 ~ 99.6%, 95.1 ~ 99.6%, 95.0 ~ 99.6% and 95.2 ~ 99.8% identities with other capripoxvirus isolates, respectively. The nucleotide sequence identities among SPPV/GanS/2/2011/China, SPPV/GanS/1/2011/China and SPPV/NingX/2009/China ranged from 99.4% to 99.6%. And, it was 99.8% between GTPV/HuB/2009/China and GS-V1. RPO30 genes of SPPV/GanS/2/2011/China, SPPV/GanS/1/2011/China, SPPV/NingX/2009/China, GTPV/HuB/2009/China and GS-V1 were also closely related to other capripoxvirus isolates (97.1 ~ 99.8%, 97.3 ~ 99.8%, 97.1 ~ 99.8%, 97.1 ~ 99.8%, 97.3 ~ 99.8%, respectively). GTPV/HuB/2009/China shared 99.8% identity with GS-V1. The sequence identities among SPPV/GanS/2/2011/China, SPPV/GanS/1/2011/China and SPPV/NingX/2009/China ranged from 99.7% to 99.8%.

### Phylogenetic analysis

**Figure 2:**
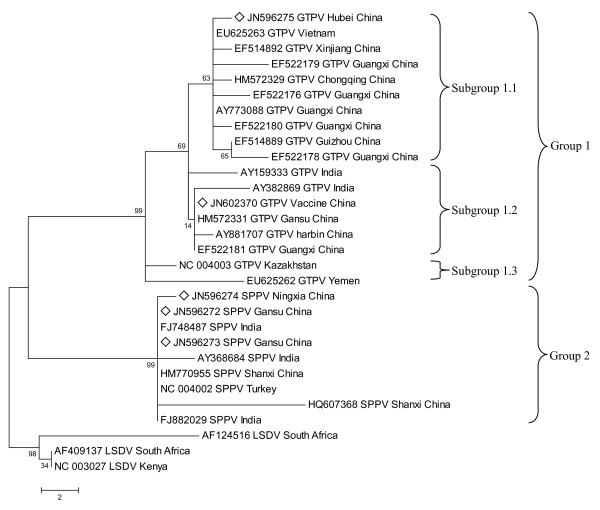
**Phylogenetic analysis of different capripoxviruses based on the nucleotide sequences of P32 gene**.

**Figure 3:**
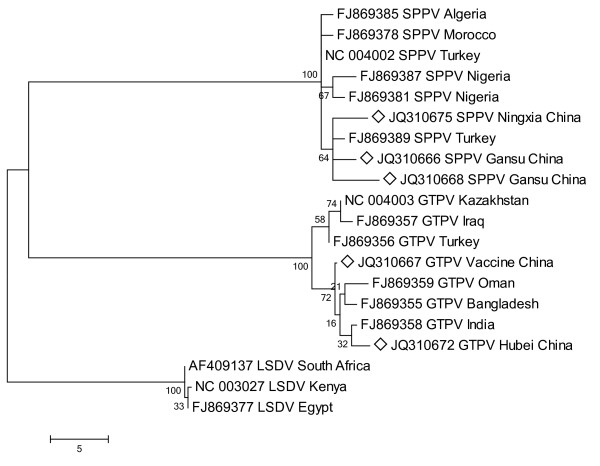
**Phylogenetic analysis of different capripoxviruses based on the nucleotide sequences of GPCR gene**.

**Figure 4:**
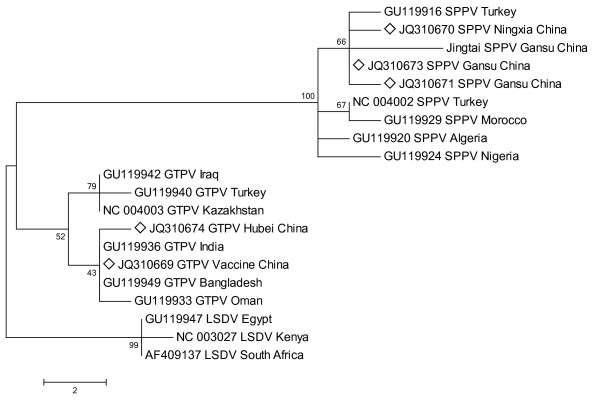
**Phylogenetic analysis of different capripoxviruses based on the nucleotide sequences of RPO30 gene**.

Phylogenetic analysis was performed using MEGA 4 with Maximum Parsimony method (Figure 2, 3, 4). The three phylogenetic trees provided good support for the three main capripoxvirus lineages: SPPV, GTPV and LSDV. SPPV/GanS/2/2011/China, SPPV/GanS/1/2011/China and SPPV/NingX/2009/China were clustered with SPPVs, while GTPV/HuB/2009/China and GS-V1 were clustered with GTPVs. P32 gene analysis showed that the lineage of GTPV strains could be roughly divided into three sub-clusters. Furthermore, GTPV/HuB/2009/China and GS-V1 (GTPV, China) belonged to separate sub-clusters respectively. However, all the SPPV strains formed a single cluster. When analyzing GPCR gene and RPO30 gene, the two phylogenetic trees showed similar topological structure. The GTPVs involved could be divided into two sub-clusters. GTPV/HuB/2009/China and GS-V1 were clustered together with GTPVs from India, Bangladesh and Oman. In SPPV lineage, all the Chinese SPPVs belonged to a separate sub-cluster, and Turkey/98 Van2 (SPPV, Turkey) was clustered together with Chinese viruses.

## Discussion

In more recent years, outbreaks of sheep/goat pox occurred in Greece in 2008, in Mongolia in 2008 and 2009, in Kazakhstan and Azerbaijan in 2009, in Taiwan China in 2008 and 2010 [1], and in India in 2011 [11]. Listed as Class A animal diseases, goat pox and sheep pox are endemic in several parts of China. They reduce the productive potential of the sheep and goat industries, and limit the development of intensive feedlot system and the improvement of ovine variety breeding. According to Chinese official data and published literatures, in the last decade, most of the case reports were from northwest China (mainly involving Ningxia Hui Autonomous Region, Gansu Province and Qinghai Province), followed by central and southern China [12].

In the present study, four field strains were isolated from three provinces of China in 2009 and 2011. All the four cases showed pyrexia, as well as macules and papules in the skin. Some cases showed multifocal necrotic lesions in internal organs including lung (SPPV/NingX/2009/China) and gastrointestinal tract (SPPV/GanS/1/2011/China). Among them, the most serious case was caused by SPPV/NingX/2009/China. In 48 Small-Tail Han Sheep, all of them got ill, and as a result, 23 sheep (including 11 lambs) were dead. All animals showed pyrexia (39.7 ~ 41.2°C), and skin nodules could be observed in most of them. Pustular lesions were also observed on the surface of lungs of dead animals. This outbreak was attributed to a stud ram from other province. GTPV/HuB/2009/China was collected from a flock of crossbred of Boer Goat with local goat (n = 68). The morbidity was 76.5% and 19 goats (including 12 lambs) were dead. According to the information provided by the owner, the goat flock had never been vaccinated. SPPV/GanS/1/2011/China and SPPV/GanS/2/2011/China were both collected from Gansu province, an important province of husbandry with broad and various kinds of natural meadow. Both two outbreaks were associated with nondescript local breeds of sheep, the morbidity was 71.3% (n = 87) and 61.5% (n = 109), respectively. SPPV/GanS/1/2011/China resulted in two ewes abortion and 21 sheep dead, while SPPV/GanS/2/2011/China led to six deaths.

So far, the simple and efficient detection techniques such as enzyme-linked immunoabsorbent assay (ELISA) for capripox haven't been popularized in China, besides, conventional serological methods cannot distinguish SPPV and GTPV. So the most frequently used technique is still PCR test. Herein, we analyzed the complete ORFs of P32 gene, GPCR gene and RPO30 gene of four natural isolates and a vaccine strain by comparing them with other capripoxviruses. Analyses of the three genes confirmed the five viruses as three SPPVs and two GTPVs consistently, which were accordant to their host species. Based on alignment and phylogenetic analysis of P32 gene, the five strains were distributed into two separate groups (Figure 2). In Group 1, GTPV/HuB/2009/China belonged to Subgroup 1.1, while GS-V1 in Subgroup 1.2. We found that most of the members in Subgroup 1.2 were vaccine strains. If we classify Pellor (GTPV, Kazakhstan) and Sana'a/1983 (GTPV, Yemen) as Subgroup 1.3, it is obvious that GTPVs of Subgroup 1.1 and Subgroup 1.2 were all from eastern and southern Asia, and Chinese strains were clustered in both Subgroup 1.1 and Subgroup 1.2. When referring to the alignment of amino acid residues, we found that GTPVs of Subgroup 1.3 had an asparagine at position 46 same as SPPVs and LSDVs. Nevertheless, the rest GTPVs replaced it with a lysine. Whether the lysine at 46 is a signature of GTPVs from eastern and southern Asia needs more evidences yet. GTPV/GanS/2009/China and s7 (GTPV, China) had peculiar two extra lysines encoded by "AAAAAA", two P32 sequences of which were 100% identical. However, s7 was a vaccine strain [13], while GTPV/GanS/2009/China was a field GTPV isolated from sheep [14]. Whether the two strains were derived from the same progenitor or the vaccine strain s7 recovered its virulence through mutation or recombination, which has not been verified. In addition, Venkatesan G *et al*. reported that an Indian GTPV isolate was closely related to s7 (GTPV, China) and Uttarkashi (GTPV, India) according to phylogenetic analysis [15]. Uttarkashi and s7 are both currently used GTPV vaccine strains. The cases mentioned above may incur some debates on the security of current attenuated live vaccines against capripoxvirus so as to urge the development of new generation vaccines.

Based on the phylogenetic analysis of GPCR gene and RPO30 gene, the five viruses were divided into two clades coincidently in both two phylogenetic trees (Figures 3, 4). It is obvious that there is a close relationship among Chinese SPPVs as they are clustered together on a separate sub-cluster. Turkey/98 Van2 (SPPV Turkey) is more closely related to Chinese viruses unlike another Turkish SPPV TU-V02127 and other SPPVs from African countries. In GTPV lineage, there are two sub-clusters. At present, the informations of GPCR gene and RPO30 gene of Chinese GTPVs are limited, but they may indicate a close relationship between Chinese GTPVs and other isolates from southern Asian countries. Besides, GTPVs form central and western Asia maybe exist closer genetic relatedness, which is also supported by P32 analysis. Comparing to GPCR gene and RPO30 gene, P32 gene is more suitable for epidemiological research on Chinese isolates because of its available abundant information. Nevertheless, just one gene is not enough to elucidate the genetic relatedness of capripoxviruses for their huge size genomes. Multi-genetic and genomic level study will greatly improve the accuracy and contribute to Chinese capripoxvirus epidemiological study.

Although the sequences of P32 gene, GPCR gene and RPO30 gene can be used to differentiate SPPV, GTPV and LSDV, it is unknown that whether the informations they containing can indicate the host specificity of the viruses. In addition, Le Goff C *et al*. overthrew previous identification of Arabian GTPV (FJ869360) and Soudanese GTPV (FJ869361) by GPCR gene analysis [16]. After all, there have been several case reports about cross infection caused by GTPV and SPPV. So, it's very necessary to establish a clear-cut standard to define SPPV and GTPV. Anyway, as genetic signatures, P32 gene, GPCR gene and RPO30 gene will be helpful in tracing the source of capripoxvirus and penetrating the epidemic regulation, thus, make it possible to prevent and control capripox effectively.

## Conclusion

In this study, four capripox cases were detected, and identified as three SPPV infections and one GTPV infection. Based on sequence and phylogenetic analysis, this paper elucidated the genetic relationship in Chinese SPPVs/GTPVs, as well as viruses isolated from other regions. On the whole, the results of this study will be useful to reveal the spread trend of SPPV and GTPV, particularly for northwestern China, and provide the theoretical references for controlling and prevention of capripox.

## Materials and methods

### Samples

Tissue samples of SPPV/GanS/1/2011/China and SPPV/GanS/2/2011/China were collected from diseased sheep in 2011 in Gansu Province, China. More specifically, SPPV/GanS/1/2011/China was collected from Dingxi City, and SPPV/GanS/2/2011/China was from Jiuquan City; Tissue sample of SPPV/NingX/2009/China was collected from a diseased sheep in 2009 in Wuzhong City, Ningxia Hui Autonomous Region, and GTPV/HuB/2009/China was from a diseased goat in 2009 in Suizhou City, Hubei Province. GS-V1 was collected from a GTPV vaccine, which was purchased from China Animal Husbandry Industry Co. Ltd.. The GTPV vaccine strain was preserved by China Veterinary Culture Collection Center with the code CVCC AV41.

### Treatment of samples, identification of capripoxviruses by PCR

Papules (or scabs, formed over the lesions) on ovine lips were collected respectively and suspended in 0.01 M phosphate-buffered saline (PBS), pH 7.4 with 10% concentration (W/V). After this, the suspension was triturated into homogenate. According to the manufacturer's instruction, DNA was extracted using the EZgene™ Viral DNA/RNA Minkprep kit (Biomiga, USA). Treated samples were subjected to PCR test to diagnose capripoxvirus infection. Involved primers as follows were synthesized by Invitrogen Inc. (Shanghai, China): CPV-1 (5'-AAA GAG GTA AAA AGT TCT ATT G-3') and CPV-2 (5'-TAA GAA AAA TCA GGA AAT CTA TG-3'). A recombinant plasmid pGEM-T-P32 containing the complete P32 gene was used as positive control, and deionized water was used as negative control.

### Viral isolation and electron microscope examination

PCR-positive samples were treated by antibiotics and added to Vero cells. Then, viruses were passed in Vero cells. After amplification cultivating, viruses were concentrated and purified by discontinuous sucrose gradient centrifugation. Suspended materials of each layer were examined by transmission electron microscope with the help of Experimental Apparatus Center of Gansu Agricultural University.

### Cloning and sequencing of P32 genes, GPCR genes and RPO30 genes

Extracted viral DNA previously was used to amplify the P32 gene, GPCR gene and RPO30 gene. The first set of primers: P32-1 (5'-ATG GCA GAT ATC CCA TT-3') and P32-2 (5'-TTA CCA CAG GCT ATT AGA AG-3') amplify the 1181 bp fragment containing the complete P32 ORF. The second set of primers: GPCR-1 (5'-TTT ATC AGC ACT AGG TCA TTA TCT-3') and GPCR-2 (5'-TAT CAC TCC CTT CCA TTT TTA T-3') amplify the 1684 bp fragment containing the complete GPCR ORF. The third set of primers: RPO30-1 (5'-CTC TGT TCC AAA CTA AAT CAT-3') and RPO30-2 (5'-TTT TTG TAT TAC CAA TTT CTG-3') amplify the 1385 bp fragment containing the complete RPO30 ORF. All of the primers were designed by OLIGO 7 (version 7.54) based on the previously published sequences of GTPV isolate Pellor (NC_004003) and synthesized by Invitrogen Inc. (Shanghai, China). Sample 5 was treated with the same procedures above, which was taken from a GTPV vaccine.

The PCR reaction system (total volume of 100 μl) contained 20 ng of extracted DNA, 20 μl 5 × PS buffer, 1 μl PrimeSTAR^® ^DNA polymerase (TAKARA, China), 8 μl 2.5 mM dNTP, 63 μl Nuclease-free water, 0.20 μM of each primer. DNA amplification was performed by Personal Thermal Cycler (BIO-RAD, USA). Thermal cycling parameters were: initial denaturation at 98°C for 10 sec, then 35 cycles of: denaturation at 98°C for 10 sec, annealing at 47°C for 20 sec, and extension at 72°C for 120 sec, followed by final extension at 72°C 20 min.

Reaction products of all isolates were analyzed by 1% agarose gel electrophoresis with ethidium-bromide staining. The expected amplicons were obtained and purified by AxyPrep™ DNA Gel Extraction kit (Axygen, USA). Subsequently, all the 15 amplicons were sent for sequencing by Invitrogen Inc. (Beijing, China). The coding sequences of P32 gene, GPCR gene and RPO30 gene of the five strains were submitted to GenBank with accession numbers in Table 1. Sequence information was analyzed by MegAlign module of Lasergene package (DNASTAR Inc. USA).

**Table 1:** Detailed information of Capripoxvirus strains used in the study

Accession number			Species of capripoxvirus	Strain	**Country of isolation**^**a**^
**P32 gene**	**GPCR gene**	**RPO30 gene**			

JN596272	JQ310666	JQ310671	SPPV	SPPV/GanS/2/2011/China	Gansu, China

JN596273	JQ310668	JQ310673	SPPV	SPPV/GanS/1/2011/China	Gansu, China

JN596274	JQ310675	JQ310670	SPPV	SPPV/NingX/2009/China	Ningxia, China

JN596275	JQ310672	JQ310674	GTPV	GTPV/HuB/2009/China	Hubei, China

JN602370	JQ310667	JQ310669	GTPV	GS-V1^b^	Gansu, China

NC_004002	NC_004002	NC_004002	SPPV	TU-V02127	Turkey

AY368684			SPPV	Rumanian Fanar^c^	India

HQ607368			SPPV	AV40	Shanxi, China

HM770955			SPPV	Shanxi	Shanxi, China

FJ882029			SPPV	Pune-08	India

FJ748487			SPPV	Makdhoom	India

	FJ869389	GU119916	SPPV	Turkey/98 Van2	Turkey

	FJ869387	GU119924	SPPV	Nigeria/77	Nigeria

	FJ869385	GU119920	SPPV	Algeria/93 Djelfa	Algeria

	FJ869381		SPPV	Vaccine Nigeria/99 ^b^	Nigeria

	FJ869378	GU119929	SPPV	Morocco vaccine strain ^b^	Morocco

			SPPV	Jingtai^d^	Gansu, China

NC_003027	NC_003027	NC_003027	LSDV	Neethling 2490	Kenya

AF409137	AF409137	AF409137	LSDV	Neethling Warmbaths LW	South Africa

AF124516			LSDV	Neethling^b^	South Africa

	FJ869377	GU119947	LSDV	Egypt/89 Ismalia	Egypt

HM572329			GTPV	GPV/ChongQ/2009/China	Chongqing, China

EF522179			GTPV	s5	Guangxi, China

EF522176			GTPV	s1	Guangxi, China

AY382869			GTPV	Uttarkashi ^c^	India

EF522178			GTPV	s4	Guangxi, China

NC_004003	NC_004003	NC_004003	GTPV	Pellor	Kazakhstan

AY773088			GTPV	Liujiang/2003	Guangxi, China

AY881707			GTPV	vaccine strain^b^	Harbin, China

EF514892			GTPV	Y	Xinjiang, China

EF514889			GTPV	LD	Guizhou, China

EF522180			GTPV	s6	Guangxi, China

AY159333			GTPV	Mukteswar	India

EF522181			GTPV	s7^b^	Guangxi, China

HM572331			GTPV	GPV/GanS/2009/China	Gansu, China

EU625263			GTPV	Ninh Thuan/2005	Vietnam

EU625262			GTPV	Sana'a/1983	Yemen

	FJ869359	GU119933	GTPV	Oman/84	Oman

	FJ869357	GU119942	GTPV	Iraq/61 Gorgan	Iraq

	FJ869355	GU119949	GTPV	Bangladesh/86	Bangladesh

	FJ869358	GU119936	GTPV	India/83	India

	FJ869356	GU119940	GTPV	Turkey/98 Denizli	Turkey

Note: a: Chinese isolates were detailed to province location; b: Vaccine strain; c: Attenuated strain; d: Sequence information was derived from an article [17]; The sequences of JN596272, JN596273, JN596274, JN596275, JN602370, JQ310666, JQ310668, JQ310675, JQ310672, JQ310667, JQ310671, JQ310673, JQ310670, JQ310674 and JQ310669 were generated in the present study.

### Phylogenetic analysis

The nucleotide sequences of P32 gene, GPCR gene and RPO30 gene of other capripoxvirus strains were obtained from the GenBank database using online BLAST program on NCBI. Multiple-alignment of these sequences was performed by MEGA 4 (version 4.0) with ClustalW method. Then, MEGA 4 was used to perform phylogenetic analysis with Neighbor-Joining (NJ), Maximum Parsimony (MP) and Minimum Evolution (ME) [18,19]. Reliability of phylogenetic trees was tested by bootstrap analysis with 1000 replicates [20]. All the methods resulted in similar topological trees. The consensus MP trees were reconstructed and shown as rectangular trees in Figure 2, 3, 4. For further confirmation, three other phylogenetic trees were reconstructed using Phylip v3.69. In brief, the sequences were aligned by ClustalW2 [21]. The phylogenetic trees were reconstructed with MP method, calculated using the modules SEQBOOT, DNAPARS and CONSENSE of Phylip v3.69 package. The phylogenetic trees were displayed by Tree-View program [22].

## Competing interests

The authors declare that they have no competing interests.

## Authors' contributions

ZJ, HJ and TZ conceived the study. All authors participated in field work, laboratory work, manuscript proof reading and review. TZ and HJ performed the data analysis. TZ wrote the paper. All authors have read and approved the final manuscript.
